# Detection of Transient Bacteraemia following Dental Extractions by 16S rDNA Pyrosequencing: A Pilot Study

**DOI:** 10.1371/journal.pone.0057782

**Published:** 2013-03-04

**Authors:** Alfonso Benítez-Páez, Maximiliano Álvarez, Pedro Belda-Ferre, Susana Rubido, Alex Mira, Inmaculada Tomás

**Affiliations:** 1 School of Medicine and Dentistry, Santiago de Compostela University, Santiago de Compostela, Spain; 2 Genomics and Health Department, Centre for Advanced Research in Public Health (CSISP), Valencia, Spain; 3 Department of Microbiology, University Hospital Complex, Institute for Biomedical Research, Vigo, Spain; Charité-University Medicine Berlin, Germany

## Abstract

**Objective:**

The current manuscript aims to determine the prevalence, duration and bacterial diversity of bacteraemia following dental extractions using conventional culture-dependent methods and 16S rDNA pyrosequencing.

**Methods:**

The study group included 8 patients undergoing dental extractions under general anaesthesia. Peripheral venous blood samples were collected at baseline, 30 seconds and 15 minutes after the dental extractions. Blood samples were analysed for bacteraemia applying conventional microbiological cultures under aerobic and anaerobic conditions as well as pyrosequencing using universal bacterial primers that target the 16S ribosomal DNA gene.

**Results:**

Transient bacteremia was detected by culture-based methods in one sample at baseline time, in eight samples at 30 seconds, and in six samples at 15 minutes after surgical procedure; whereas bacteraemia was detected only in five blood samples at 30 seconds after dental extraction by using pyrosequencing. By applying conventional microbiological methods, a single microbial species was detected in six patients, and *Streptococcus viridans* was the most frequently cultured identified bacterium. By using pyrosequencing approaches however, the estimated blood microbial diversity after dental extractions was 13.4±1.7 bacterial families and 22.8±1.1 genera per sample.

**Conclusion:**

The application of 16S rDNA pyrosequencing underestimated the prevalence and duration of bacteraemia following dental extractions, presumably due to not reaching the minimum DNA required for PCR amplification. However, this molecular technique, unlike conventional culture-dependent methods, revealed an extraordinarily high bacterial diversity of post-extraction bacteraemia. We propose that microorganisms recovered by culture may be only the tip of an iceberg of a really diverse microbiota whose viability and potential pathogenicity should be further studied.

## Introduction

Bacteraemia is defined as the presence of bacteria in blood. A feature that is unique to the oral bacterial biofilm, particularly the subgingival plaque is its close proximity to a highly vascularised milieu. Consequently, any disruption of the natural integrity between the biofilm and the subgingival epithelium, which is at most about 10 cell layers thick, could lead to a bacteraemic state [1]. For several decades, the haematogenous spread of bacteria from the oral cavity has been considered a decisive factor in the pathogenesis of 10% to 15% of cases of infective endocarditis (IE); oral infections or certain dental procedures may therefore carry a significant risk [2]. In addition to its possible role in the onset of IE episodes, oral-derived bacteraemia has attracted particular interest in the past two decades given its possible involvement in the progression of atherosclerosis and its consequent implication in the development of ischaemic disease; however, the mechanism of action has not yet been fully elucidated [3]–[5].

A recent review of the literature revealed a prevalence of transient bacteraemia after dental extractions (BDE) that varies between 30% and 76% in children and between 58% and 100% in adults [6]. There are several culture-based microbiological procedures for the analysis of blood recovered after dental extractions. Procedures such as quantitative methods [7], semi-quantitative methods (lysis-centrifugation technique or lysis-filtration technique) [8], [9] or qualitative methods using automated reading systems based on the detection of the CO_2_ produced by bacterial growth [10]–[12] have been used with the aim of detecting major bacterial species in transitory bacteraemias. Nevertheless, after reviewing published data of oral-derived bacteraemia significant differences were detected between studies in relation not only to the microbiological procedures applied, but also to the transport and culture media, the atmosphere and incubation times used, and the characteristics of the isolates phenotypic identification process [6], [13]. All these factors could affect bacterial isolation and identification (particularly of fastidious oral bacteria), and it has therefore been stated that “oral bacteria recovered from blood by culture are probably only part of those present” [5]. As a result, recently developed methods for the specific detection and identification of microorganisms, particularly polymerase chain reaction (PCR) techniques, have renewed the interest in this field, as shown by the recent work performed by several authors [14]–[18]. However, only a few studies have compared conventional culture methods and 16S rDNA PCR for detection of bacteraemia after different oral procedures [14], [17]. The aim of the present study was to determine the prevalence, duration and bacterial diversity of bacteraemia following dental extractions using the conventional culture-based microbiological techniques and the 16S rDNA pyrosequencing approach, which is the first time that is applied to this kind of samples.

## Patients and Methods

### Selection of the Study Group and Clinical Examination

The study group consisted of 8 patients who, for behavioural reasons (autism, cerebral palsy, learning disabilities, hyperactivity, phobias, etc.), underwent dental extractions under general anaesthesia in the Santiago de Compostela University Hospital (Spain). The following exclusion criteria were applied: patients who had taken antibiotics in the 3 months prior to the study (including antibiotic prophylaxis for the surgical procedure in the present series), routine use of oral antiseptics, patients suffering from any type of congenital or acquired immunodeficiency, and any other disease which could predispose to infections or bleeding.

A single trained examiner performed an intraoral examination after nasotracheal intubation and before carrying out the dental extractions gathering information on: dental plaque accumulation (oral hygiene index of Greene and Vermillion simplified) [19], calculus accumulation (calculus index of Ramfjord) [20], presence of gingival bleeding (gingival index of Löe and Silness) [21], depth of periodontal pockets, grade of dental mobility (Ramfjord index of dental mobility) [20], and number of decayed teeth (including remaining roots). Periodontal disease was evaluated using previously established diagnostic criteria [22]. The number of teeth extracted was also recorded.

### Ethics Statement

The project was approved by the Clinical Research Ethical Committee of Galicia (2008/202). Written informed consent for participation in the study was obtained from the patients or their legal representatives in all cases.

### Collection of Subgingival Samples

Before the dental manipulation, subgingival samples were collected from the teeth to be extracted (in 2 locations -vestibular and palatine/lingual- and 2 consecutive paper points at each location, avoiding contamination by supragingival plaque and saliva) to determine the prevalence and proportion of oral bacteria present in the gingival sulcus (or periodontal pocket). To preserve DNA integrity and avoid changes in phylotype abundance [23], subgingival samples were stored in phosphate buffer solution at −80°C until needed for subsequent 16S rRNA pyrosequencing analysis.

### Collection of Blood Samples

To determine the prevalence of BDE, peripheral venous blood samples (10 ml) were collected from each patient at baseline (after nasotracheal intubation and before local anaesthetic injection with articaine and adrenaline), 30 seconds and 15 minutes after the final dental extraction. The collection, handling and transport of samples for blood culture were performed according to the recommendations of the Spanish Society of Infectious Diseases and Clinical Microbiology [24]. For 16S rDNA pyrosequencing, blood samples were inoculated in 6 ml vacutainer tubes containing citrate (Becton Dickinson and Company) and were stored at −80°C.

### Conventional Microbiological Analysis of Blood Cultures

Bottles with the aerobic and anaerobic culture media (BactecPlus, Becton Dickinson and Company, Sparks, MD, USA) into which the blood samples were inoculated were processed in the Bactec 9240 (Becton Dickinson). A Gram stain was performed on each positive blood culture. The positive blood cultures in the aerobic media were subcultured on blood agar and chocolate agar in an atmosphere of 5–10% CO_2_, and on MacConkey agar under aerobic conditions. The same protocol was used for positive anaerobic blood cultures, though also including subculture on Schaedler agar incubated in an anaerobic atmosphere. All bacteria isolated were identified using the battery of biochemical tests provided by the Vitek system (bioMerieux Inc., Hazelwood, Missouri, USA) for Gram-positive bacteria, *Neisseria* spp./*Haemophilus* spp. and obligate anaerobic bacteria. The *Streptococcus viridans* were classified into 5 groups: *mitis, anginosus, salivarius, mutans,* and *bovis* by applying the Ruoff criteria [24]–[26].

### DNA Isolation

DNA from transient free living bacteria present in blood samples was isolated using the MolYsis Complete5 kit (Molzym GmbH & Co.KG) for enrichment of bacterial DNA following the manufacturer recommendations. This extraction method eliminates freely-suspended DNA and human DNA by a DNAse treatment and a human-specific lysis, obtaining DNA almost exclusively from free bacterial cells [27]. Subgingival samples were also processed using same extraction procedure to avoid bias amplification by extraction protocol.

### PCR Amplification and Pyrosequencing

Variable V1, V2, and V3 regions of the 16S rDNA were amplified with the universal eubacterial primers 27F (5′-AGAGTTTGATCMTGGCTCAG-3′) and 533R (5′-GCCTTGCCAGCCCGCTCAGGC-3′) using the high-fidelity AB-Gene DNA polymerase (Thermo Scientific). A first PCR reaction was set up with an annealing temperature of 54°C and 20 cycles of amplification with the aim to minimize PCR bias amplification [28]. A nested amplification was performed using the purified PCR product from the first reaction as a template, with the same reaction conditions, in which the universal primers were shifted 3 bp towards the 3′ end and modified to contain the pyrosequencing adaptors A and B, an 8bp “barcode” specific to each sample [29]. Barcodes were different in at least 3 nucleotides from each other to avoid mistakes in sample assignments. Three replicates of secondary PCRs were performed per sample and pooled. PCR products were purified using the High Pure PCR Product Purification Kit (Roche). The PCR products from the primary PCR negative controls were used as templates for the secondary PCR negative control. The final DNA per sample was measured by fluorescent method (PicoGreen® Invitrogen) and the different PCR samples from gingival sulcus (or periodontal pockets) and blood were mixed in equimolar proportions. The pool of tagged PCR samples was further concentrated by using Amicon 100K filters (Millipore) up to a concentration higher than 100 ng/ul. PCR products were pyrosequenced from the forward primer end only using a 454 GS-FLX pyrosequencing platform with Titanium chemistry (Roche) at the Genomics and Health Unit at the Center for Public Health Research (CSISP) in Valencia, Spain. One eighth of a plate was used for sequencing the pool of PCR amplicons. After the nested-PCR 16S rDNA amplification followed by pyrosequencing we obtained a mean of ∼1860 reads per sample (ranging from 584 to 6822 reads) with a mean length of 350 nucleotides.

### Sequence Analysis

Reads with an average quality value lower than 20 and/or with more than 4 ambiguities in homopolymeric regions in the first 360 flows were excluded from the analysis. Only reads longer than 150 bp were considered, and chimeric sequences were filtered out using the software Bellerophon [30]. Sequences were assigned to each sample by the 8-bp barcode and analyzed with the Ribosomal Database Project (Release 10, Update 27) classifier [31]. Each read was assigned a phylum, class, family and genus, as long as the taxonomic assignment was unambiguous within an 80% confidence threshold, which has been estimated to taxonomically assign reads with over 98% accuracy at the genus level. Larger sets of unclassified bacteria were manually assigned using a Blastn search against non-redundant nucleotide database at GenBank. To estimate total diversity, sequences were clustered at 97% nucleotide identity over 90% sequence alignment length using the Mothur software v1.21.0 [32]. For this analysis, sequences over 97% identical were considered to correspond to the same Operational Taxonomic Unit (OTU), representing a group of reads which likely belong to the same species [33].

## Results

### Characteristics of the Study Group

The study group comprised five males and three females, with an averaged age of 30.3±7.6 years (range 21–38 years). Seven patients had Grade ≥2 supragingival plaque accumulation and Grade ≥2 gingival inflammation. Grade ≥1 tooth mobility was observed in four patients. A previous diagnosis of generalized chronic periodontal disease was made in four patients. The mean number of dental extractions was 3.87±3.83.

### Diversity of Subgingival Microbiota by Pyrosequencing

**Figure 1:**
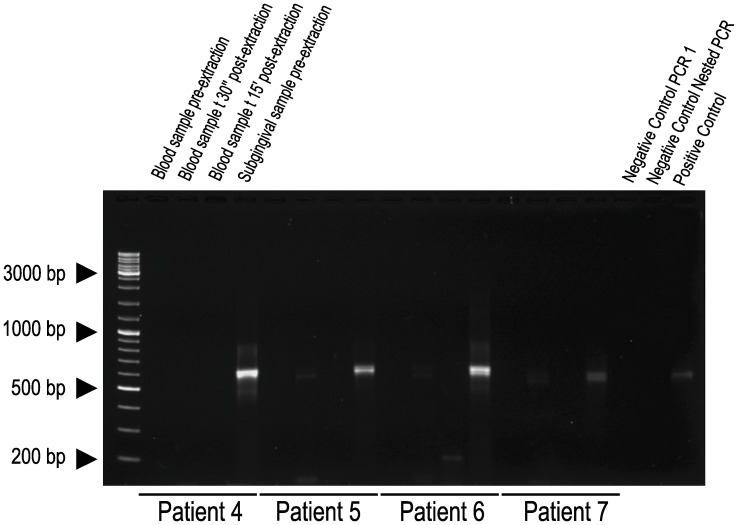
Gel of electrophoresis of 16S rDNA amplified by nested-PCR. A set of four samples from four different patients are shown according to time collection: blood sample before dental extraction, blood sample 30 sec after dental extraction, blood sample 15 min after dental extraction, and subgingival plaque sample of teeth to be extracted. The last three lanes on the right show the respective negative and positive controls. PCR product expected is ∼560bp in length.

**Figure 2:**
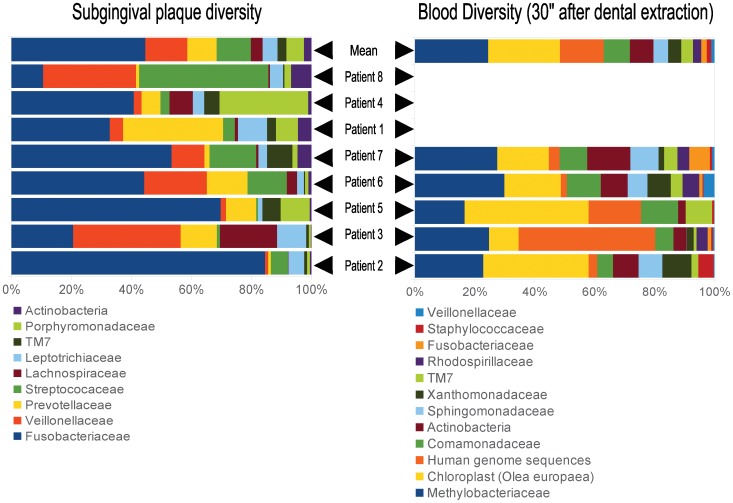
Distribution and diversity of bacterial families. Only families present in a percentage higher than 1% of the total bacterial population detected in subgingival and blood samples of each one of the 8 patients are shown.

Paper points were collected from molars (62.5%), premolars (18.7%), incisors (12.5%) and canines (6.3%). Half of subgingival samples derived from gingival sulcus and the other half from periodontal pockets. By applying 16S rDNA PCR amplification (Figure 1) followed by pyrosequencing, the common bacterial diversity at family level could be identified in subgingival samples. Thus, the most probable repertoire of organisms able to enter into bloodstream was determined, being Fusobacteriaceae, Veillonellaceae, Prevotellaceae, Streptococaceae, Porphyromonadaceae, Leptotrichiaceae, Lachnospiraceae, TM7, and Actinobacteria, present in more than 1% in subgingival samples from all patients (Figure 2).

### Prevalence and Duration of Bacteraemia following Teeth Extraction

By applying conventional microbiological culturing techniques, bacteremia was detected in 1 sample at baseline time, in all blood samples at 30 seconds after the dental extractions, and in six blood samples collected at 15 minutes after finishing the surgical manipulation. By applying 16S pyrosequencing, bacteraemia was detected only in five blood samples at 30 seconds after dental extractions (see Figure 1).

### Bacterial Diversity Associated to Bacteraemia

**Figure 3:**
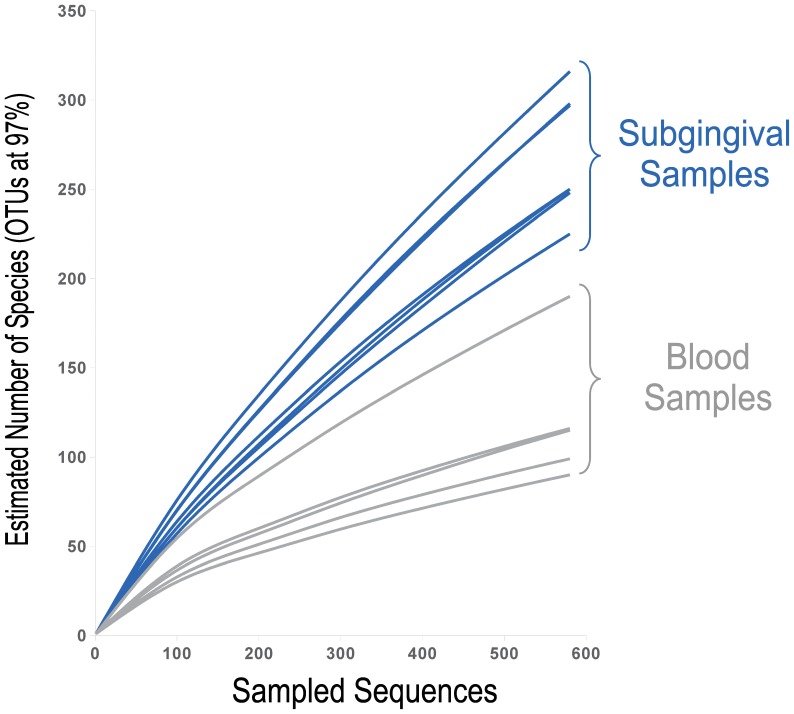
Rarefaction curves which relate the sequencing effort to the number of putative species in the samples. Blue curves represent diversity in subgingival samples whereas Grey curves show diversity in blood samples where transitory bacteraemia was detected. Rarefactions curves are uniformly presented from a sub-set of sequences randomly selected from each sample dataset. The number of species-level phylotypes was calculated by clustering sequences at 97% sequence identity, which has been determined as the threshold for species boundaries [33], [53].

A poor diversity was evidenced in all samples tested by culture. Specifically, single-species cultures from blood were detected in six patients whereas two remaining samples showed two bacterial genera. We identified a total of 18 isolates from positive blood cultures (one isolate in blood cultures obtained at baseline samples, nine from those obtained at 30 seconds after the dental extractions, and eight from those obtained at 15 minutes after ending the surgical procedure). Thirteen out of the eighteen isolates were identified as *Streptococcus viridans* (nine mitis group and four salivarius group), two isolates as *Peptoniphilus assaccharolyticus*, two isolates as *Gemella* spp., and one as *Actinomyces* spp. By applying 16S rDNA pyrosequencing we were able to detect a higher diversity in all samples where transitory bacteraemia was observed, with a mean of 13.4 bacterial families and 22.8 bacterial genus per sample. The number of species in blood samples can be observed in rarefaction curves which relate the sequencing effort to the number of putative species in the samples (Figure 3). For the same number of sequences analyzed, the diversity in 30′′ blood samples appears to be about half of that found in subgingival samples, and the shape of the curves indicate that numerous species (>50 species) are likely to be present in blood.

Unexpectedly, the most frequent reads from 16S rDNA pyrosequencing were assigned to the Methylobacteriaceae family in all samples where bacteraemia was detected (21.5% of reads on average). Other frequent bacterial groups present in blood samples are the Comamonadaceae family and the Actinobacteria class of bacteria (this last composed by Actinomycetaceae, Corynebacteriaceae, Propionibacteriaceae, and Micrococcaceae families) found approximately in 7% of 16S rDNA reads amplified and pyrosequenced. Other relevant groups present in blood in more than 1% of reads on average and being associated to the subgingival microbiota are the Fusobacteriaceae, Veillonellaceae, and TM7 families of bacteria.

## Discussion

Several molecular techniques have been applied for the specific detection and identification of microorganisms present in the bloodstream after dental procedures [14]–[18]. The results presented in here, to the best of our knowledge, the first data on the analysis of transient bacteraemia after dental extractions by conventional microbiological techniques and pyrosequencing.

### Considerations to Detect Oral-derived Bacteraemia by Molecular Techniques

In general terms, “bacteraemia” refers to live microorganism in the bloodstream and PCR does not discriminate between live and dead bacteria [14]. In addition to this major disadvantage, the high sensitivity of the PCR technique could be problematic, as any contamination occurring during blood specimen collection (e.g. the skin during venepuncture), or in laboratory processing, could easily lead to detect false positives [34]. In the present study, despite a high risk of contamination resulting from nested PCR approach, no amplification signals were observed in negative controls from primary and secondary PCRs (Figure 1). In addition, the negative amplification of bacterial 16S rDNA from blood samples before dental extraction further supports the absence of artifacts in the results.

However, the lower sensitivity of 16S pyrosequencing observed in the present study, this is consistent with previous reports that have highlighted specific problems when using PCR to detect bacteraemias, including oral bacteraemia [17]. Essentially, low sample volume [35] and bacterial load [36] result in a loss of PCR sensitivity. Data derived from flow cytometry indicate that at least 5,000 bacterial cells are needed for obtain a PCR product (G. d’Auria, personal communication). Therefore, our contrasting results of BDE detected by culture-based and molecular methods could be explained by bacterial load given that similar blood sample volumes were used on both analyses. At this respect, quantitative analysis such a qPCR approach should be performed to establish detection threshold of our methodology. Molecular methods are also affected by amplification bias dependent of primer pairs used to amplify [37]. In the present study, variable V1, V2, and V3 regions of the 16S rDNA were amplified with the universal eubacterial primers 27F/538R. Although these primers are able to detect most bacterial species, some studies have reported that rDNA from *Actinomyces* and other high G+C content species are poorly detected using universal primers [38]. The latter would explain some PCR negative results from samples with positive presence of *Actinomyces* spp. as detected by culture methods.

To avoid a potential decrease in PCR sensitivity because sample storage [39], PCR inhibitors from blood [40], and DNA degradation [41] a bacterial DNA isolation protocol was used which removes human cells, blood-derived PCR inhibitors, and DNases prior to the enrichment and lysis of bacterial cells (see methods). In this respect, DNA extraction protocols have to be taken into account as a critical issue during detection of oral-derived bacteraemias because the performance of the methodology influences the bacterial load detected in the sample. Our results showed a much higher level of PCR sensitivity in comparison with previous reports where prevalence of bacteraemia detected by molecular methods do not exceed 30% [14], [17], [42]. This is probably the result of using a more specialized DNA extraction protocol which better preserves and enriches the bacterial DNA and/or the wound magnitude resulting from the dental procedure. In future studies, equal blood samples after dental procedures should be processed with different DNA isolation protocols to evaluate the performance of those methods and simultaneously to test the bacterial load obtained for a given dental procedure.

### Prevalence and Duration of Transient Bacteraemia

The study of bacterial communities in health and periodontitis status has shown notable differences in terms of composition [43]. In the present study ∼200 species are estimated to be present in gingival samples. Among them, we detected a high rate of periodontal pathogens (∼65%) essentially belonging to Fusobacteriaceae, Veillonellaceae and Prevotellaceae bacterial families, which could be justified by a high prevalence of gingival and periodontal diseases diagnosed in our patients.

While detection of bacteraemia under basal conditions was strongly associated to endotracheal intubation [44], other factors, such as oral health status, the number of dental extractions and the anaesthetic modality (general anaesthesia) could affect the prevalence and duration of BDE. On the other hand, PCR methodologies to detect oral-derived bacteraemia show lower or higher sensitivity than culture-based methods depending on the dental procedures performed before blood sampling [14], [17]. Notwithstanding, recent reports concluded that this combined strategy improves the accuracy of results obtained by blood culture methods alone [42]. In the present study, we detected a higher prevalence of post-extraction bacteraemia applying conventional culture techniques compared with 16S pyrosequencing at 30 seconds (eight samples *versus* five samples, respectively) and 15 minutes after dental extractions. In agreement with previous research [17], these findings could be assumed to be the result of a fast response of the immune system making the numbers of surviving free-living bacteria too low for a PCR detection threshold.

It has been stated that bacteraemia secondary to dental procedures is usually of low intensity. The magnitude of bacteraemia caused by a surgical dental procedure varies between 0 and 300 CFU/ml (median of the majority of series published to date, 1.7 CFU/ml) [6]. In addition, the number of bacteria entering the bloodstream after the extraction will also decrease with time, reducing the numbers of microorganisms in those samples. In this sense, the sensitivity of real-time, quantitative PCR techniques to quantify bacteraemia following dental manipulations has been limited up to now. Lockhart et al., reported that sensitivity of their molecular method was 25 colony-forming units (CFU) per polymerase chain reaction, which corresponds to 10^3^ to 10^4^ CFU per millilitre of blood, and all our samples were below this detection threshold [15]. Nevertheless, it has recently been demonstrated that real-time PCR can accurately identify microorganisms directly from positive blood culture bottles of patients with different infectious processes with the same sensitivity as culture-based methods (the two techniques were concordant for 97.8% of the bacteria) [45].

### Bacterial Diversity of Transient Bacteraemia

**Figure 4:**
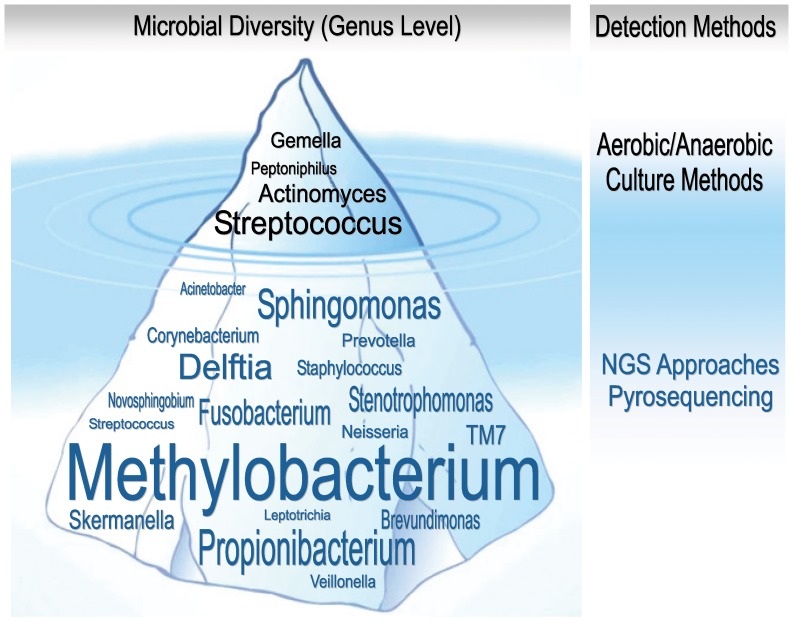
Comparative bacterial diversity (at the genus taxonomic level) detected in blood samples in this study. Genera at the top of iceberg (black labeled) are those detected by culture-based method. Genera at the submerged part of the iceberg (blue labeled) are those detected by pyrosequencing. Font size is correlated with the frequency of the respective bacteria determined by both methods.

In the present study, our results indicated that bacterial diversity obtained by culture methods is dramatically lower than obtained by 16S pyrosequencing. Although different culture media under both aerobic and anaerobic conditions were used to increase the range of microorganisms to recover, blood samples typically produced only one or two different isolates whereas the 16S pyrosequencing approach indicated 22.8+/−1.1 different bacterial genera in the blood samples. This enormous discrepancy between taxonomic identification from cultured-based and molecular methods has been previously reported [46]. In the Bahrani-Mougeot and coworkers study only 17% of isolates were identified as the same species by both methods and 55% were grouped into the same genus. Similar to our results, those authors stated that DNA sequencing resulted in a more accurate identification and more diverse population estimation of bacteria in bacteraemia after dental extractions [46]. This issue could be especially critical for those immunocompromised patients requiring oral surgical procedures and it shows that the cultured bacteria detected thus far may represent only a small percentage of the microbiota present in the bloodstream after dental extractions (see Figure 4).

Our results demonstrated that the most frequent bacterial genus found in transient bacteraemia is *Methylobacterium* spp. This genus has been reported in Intensive Care Units causing bacteraemia in inmunosupressed patients [47] and should be taken into account given its role as an opportunistic pathogen and its ability to promote biofilm formation in water lines or reservoirs [47], [48]. Given its prevalent presence in the environment and as part of the human oral microbiota [49], [50], *Methylobacterium* spp. could be a bacterium capable to avoid cell recognition at early stages of the human immune response.

The genetic relatedness between isolates from oral cavity and bloodstream samples has been analyzed by PCR techniques. Pérez-Chaparro et al., using a pulsed-field gel electrophoresis technique, confirmed the coexistence of the same bacterial clone in samples from the subgingival plaque and from peripheral blood in 16% of patients with bacteraemia following scaling and root planing [51]. Interestingly, our data indicate that the most abundant bacterial genera found in subgingival samples are found at very low proportions (0.5–1.0%) in the blood samples such as 16S rDNA sequences from *Streptococcus* spp. (0.7%), *Veillonella* spp. (0.5%), *Leptotrichia* spp. (0.4%), and *Prevotella* spp. (0.1%). However, other bacterial genera found at very low levels in the subgingival microbiota are very common in blood such as Actinobacteria (6.9%). This reversed situation could be a consequence of a very rapid immune response against the bacteria typically found in the oral cavity and for which the immune system has a strong adapted attack; these comments are in line with those reported previously by Kinane et al. [14]. To test this, DNA isolation from phagocyte immune cells present in blood after dental surgery should reveal a bacterial composition similar to that observed in subgingival samples or oral cavity as a whole. However, this issue needs further considerations being out the scope of this study.

In the present study, the taxonomic assignment of the sequences shows that the vast diversity of bacteria found in blood after dental extractions is composed mainly by fastidious species that are not able to grow in general culture conditions used for this aim. As a consequence, it is frequent to find published reports where most prevalent agents of transitory bacteraemia are those belonging to *Streptococci* and other bacterial groups [12], which could be more easily cultivable species but probably not representative of the total diversity that really enters the bloodstream. In fact, in the present study, the streptococci were found in a very low frequency with a mean of 0.7% of reads assigned to the Streptococcaceae family. Thus, the data suggest that the possibility to enter the bloodstream after a dental extraction is not restricted to the subgingival flora, but also to the whole microbiota found in different regions of the oral cavity.

After dental extractions the periodontal space, the major portal of entry of bacteria into the bloodstream [6], becomes itself a highway in which a large diversity of biomolecules and living cells can access the blood. Our data obtained by a high sensitivity molecular method supports such a scenario, as it can be observed in the prevalent detection of 16S rDNA from *Olea europaea* chloroplast in blood of patients obtained 30 seconds after dental extraction (Figure 2). This demonstrates that not only the oral microbiota is able to pass through the hematological barrier located at the periodontal space but even eukaryotic cells like those from olive oil, which contain intact cells whose DNA can be PCR-amplified [52]. As a result, intact cells or debris from food can be detected in bloodstream after dental extraction and its potential role in interfering with the immune response should be evaluated. As a consequence, it is relevant to consider potential infectious diseases caused by environmental microorganisms acquired by poor food hygiene or by non-sterile surgical utensils.

### Conclusion

The application of 16S rDNA pyrosequencing underestimated the prevalence and duration of bacteraemia following dental extractions, probably because the low bacterial load present in blood samples, thus limiting the recovery of the DNA required for PCR amplification. However, this molecular technique, unlike conventional culture-dependent methods, revealed an extraordinarily high bacterial diversity present in transient bacteraemia from oral origin. Thus, both diversity information offered by molecular methods and supported by the enormous genetic information stored in biological databases, and the better estimates of transience of bacteraemia provided by culture methods must be jointly used to better diagnose and prevent focal infections of oral origin especially in immunocompromised patients. Therefore, we propose that microorganisms recovered by culture may be only the tip of an iceberg of a really diverse microbiota whose viability and potential pathogenicity should be further studied.
